# Annoyance Judgment and Measurements of Environmental Noise: A Focus on Italian Secondary Schools

**DOI:** 10.3390/ijerph15020208

**Published:** 2018-01-26

**Authors:** Fabrizio Minichilli, Francesca Gorini, Elena Ascari, Fabrizio Bianchi, Alessio Coi, Luca Fredianelli, Gaetano Licitra, Federica Manzoli, Lorena Mezzasalma, Liliana Cori

**Affiliations:** 1 Unit of Environmental Epidemiology and Disease Registries, Institute of Clinical Physiology, National Research Council, 56124 Pisa, Italy; fgorini@ifc.cnr.it (F.G.); fabrizio.bianchi@ifc.cnr.it (F.B.); alessio.coi@ifc.cnr.it (A.C.); federica.manzoli@unimore.it (F.M.); lorena.mezzasalma@ifc.cnr.it (L.M.); liliana.cori@ifc.cnr.it (L.C.); 2 Environmental Protection Agency-Tuscany Region, 50144 Firenze, Italy; elena.ascari@gmail.com (E.A.); gaetano.licitra@arpat.toscana.it (G.L.); 3 iPool s.r.l., 56121 Pisa, Italy; luca.fredianelli@gmail.com

**Keywords:** noise, environment, children, questionnaire, annoyance index, health, annoyance, risk perception

## Abstract

The effects of noise on students’ health, well-being, and learning are of growing concern among both the general public and policy-makers in Europe. Several studies have highlighted the consequences of noise on children’s learning and performance at school. This study investigates the relationship between noise judgment in school goers aged 11–18 and noise measurements aimed at evaluating their exposure at school. For this purpose, a questionnaire was administered to 521 individuals in 28 classrooms in eight schools of four cities in Italy, with different environmental characteristics. Using a Likert-type scale, a selected set of responses related to noise generated an Annoyance Index (AI) score for each student and a classroom median score (MAI). From the noise data acquired, a global noise score (GNS) was assigned to each classroom. A higher AI was found in industrialized areas and among younger students. No significant differences in noise judgment were found by gender. A significant inverse correlation was described between MAI and GNS, thus the better the acoustic quality of the classrooms, the less the perceived noise and annoyance. The results show that noise perception and consequent disturbance are highly correlated with classroom acoustics, and confirm that annoyance represents the most widespread subjective response to noise.

## 1. Introduction

Noise is recognized as a major environmental issue, particularly in urban areas, affecting quality of life and well-being [1]. According to the World Health Organization, noise ranks second among the environmental risk factors to public health, only behind ultra-fine particulate matter (PM_2.5_) air pollution [1]. An estimated 20 million adults are subjected to noise annoyance in Europe, and a further eight million suffer sleep disturbance due to environmental noise [1,2]. Long-term exposure to environmental noise can lead to hearing loss [3], endocrine effects [4], cardiovascular diseases [5], and increased incidence of diabetes [6].

Noise influences mental, behavioral and neurological disorders that account for 3% of worldwide deaths and 10% (or 15% as forecast in 2020) of the global burden of disease [7]. Children are particularly vulnerable to both auditory and non-auditory effects of environmental noise because of their reduced ability to manage environmental stressors and less automatized cognitive functions [8,9]. The WHO estimates that cognitive impairment is responsible for 45,000 healthy life years lost every year in children aged between 7 and 19 from Western European countries [10].

Road traffic and aircraft noise at school can negatively affect the performance of school-age children, disturbing children’s attention and motivation [11,12], reading comprehension [13,14], short-term memory [15,16], and mathematical skills [17,18]. Associations were found between increased hyperactivity symptomatology in children and both road traffic noise at home [19,20] and traffic and aircraft noise at school [21,22]. In addition, there is some evidence that exposure to long-term traffic noise may increase children’s blood pressure levels [23,24].

The noise in a classroom is made up of background noise (noise from external sources plus noise transmitted from other areas of the school), in addition to internally generated noise [25]. In noisy and reverberant (i.e., when the noise takes time to fade away) classrooms, elementary school children have greater difficulty in both speech perception and listening if compared to older school-aged peers or adults [26,27,28]. Younger children appear to be less able to distinguish words against background noise due to the higher-order cognitive functions (e.g., short-term storage) involved in comprehension [27,29,30].

Recognizing the special need to protect children from the harmful effects of noise, the World Health Organization Parma Declaration [31] called upon all stakeholders to cooperate for reducing the exposure of children to noise. The call finds its completion in the aim pursued by the European Union over the past 30 years to foster the growth of a “scientific citizenship” [32]. This implies the realization of an efficient *knowledge transfer and exchange* among all the actors affected, involving the general public, as well as experts, local authorities and all the relevant stakeholders in both the natural and social sciences including risk communication. Their active involvement ensures more effective problem-solving and decision-making, pivotal in the field of environmental health risk management [33,34,35].

Risk perception, defined as an individual’s judgment of hazards which they might be exposed to, is strictly connected to decision-making in strategies aimed at reducing the impact of environmental health determinants [36]. This definition is not accepted by the whole scientific community. Environmental education at school, when the child’s personality starts to be shaped and awareness increases, plays a crucial role in the future risk perception. Children may not perceive environmental noise as a major hazard, however they may be annoyed to a degree that interferes with their tasks. A Danish survey reported that 19% of the responding children were frequently annoyed by noise during school lessons, 19% did not suffer annoyance during lessons, and 62% were annoyed only sometimes by noise [37]. In a Turkish study, high-school students perceived environmental noise as a medium-level risk factor nonetheless the questionnaire results generally showed high risk perception and awareness towards environmental factors [38]. An online questionnaire survey carried out on secondary school pupils in England identified factors (e.g., ease of hearing, annoyance) that characterized children’s perceptions of their school’s acoustical environment [39]. The proximity of schools to railways, or activities related to it, resulted in a major cause of annoyance [40].

In Italy, during the GIOCONDA project (an acronym that translates as “Young voices count in decisions on environment & health”) [41], local administrators applied an innovative methodology aimed at mobilizing knowledge and involving young people in local decision making related to environmental and health issues. The actions undertaken included the collection of air pollution and noise data; a questionnaire exploring the judgment of environmental hazards and risks, meetings with experts to understand the connection between the environment and health in their surrounding areas. These activities provided children with a knowledge basis for the production of proposals and recommendations regarding the local environment and health issues. They also provided public administrations with the opportunity to explore and include students’ judgment in the decision-making process.

The pilot phase of the GIOCONDA project was carried out in four areas in Italy with different demographic, social, and environmental conditions [42]. During this phase, a questionnaire was administered to students, and noise was monitored at school, using an innovative global indicator developed for the acoustical characterization of classrooms [43].

This article focuses on a crucial step in the management process, namely the creation of the scientific evidence where to base the *knowledge transfer*: we research the correlation between annoyance by a targeted public—the school goers—and measurement of noise in their classrooms and outside their schools

## 2. Methods

### 2.1. Project Location and Participants

In the pilot phase, the GIOCONDA project involved four Italian areas distributed along the national territory, with different socio-economic and environmental pressures: Naples, Taranto, Ravenna, Lower Valdarno Valley.

Four lower secondary schools (age 11–13) and four upper secondary schools (age 14–18) participated in the study. In each location, one of each type of school was selected. In the eight schools, 28 classes participated, including 603 students, 521 of whom completed the questionnaires (students’ distribution by school: min = 39, max = 93, mean = 65.13 ± SD = 16.86, median = 63.50).

The authorization to perform activities at school involving minors is agreed by parents signing an inform consent at the beginning of the school year. The GIOCONDA project submitted a specific request to all the parents for the use of images at school during GIOCONDA activities. Regarding the ethical issues involved in the GIOCONDA activities (questionnaires, meetings, events) we received specific directions in the framework of the advisory board, which included the responsible of Scientific Secretariat of the Research Ethics and Bioethics Committee of the National Research Council. There were two main activities regarding ethical issues, agreed by the advisory board: first, the text of questionnaires, factsheets, educational material was systematically submitted to the advisory board to monitor contents, carefully considering the risk of causing psychological trauma, fears for the future and distrust towards the authorities. Second, the topics covered by GIOCONDA activities are included in the school ordinary educational planning (both in the lower and the upper secondary schools). Moreover, we consulted the legal requirements of the Tuscany Region Ethical Committee (Pisa, the headquarter of the Institute of Clinical Physiology of the National Research Council, coordinator of Gioconda project is in Tuscany Region). The Ethical Committees in Italy are regulated at the Regional level (Ministerial Decree 8 February 2013), “Criteria for the composition and functioning of ethics committees” (Criteri per la composizione e il funzionamento dei comitati etici). The Tuscany Region Ethical Committee is competent for all the pediatric clinical research conducted on the Tuscan regional territory for clinical trials involving the use of medicines, medical devices, supplements/food products, diagnostic-therapeutic-medical-surgical procedures and biological samples.

### 2.2. Measurements of Annoyance Index

Data were collected using a self-administered printed questionnaire (with the teachers’ support, if necessary) completed in the classroom. The questions, organized into different sections, were designed to investigate the awareness of the presence of environmental disturbance, the risk perception related to air pollution and noise, and behaviors related to environmental protection. A score to estimate perceived noise, using the index described and validated in this paper, was used to examine student responses to the questionnaire about noise.

#### 2.2.1. Noise Related Questions

Students were asked to express their level of concern regarding a series of issues. The following are part of the noise-related questions administered. The questions here considered were about noise at school, and we then used the answers connected to noise measurement, done in the classroom. Even if the difference is not negligible, we think that the answer can be used in this way, because in the school we involved in the GIOCONDA project the students used to spend their time at school in the same classroom (Table 1).

#### 2.2.2. Annoyance Index

The questions reported above were used to estimate an individual Annoyance Index (AI) derived from the one previously developed [44]:

$$\mathrm{AI}=\frac{\sum_{i}^{k}n_{i}\pi_{i}}{N\cdot \left(k\right)}$$

where: *n_i_* represents the absolute frequency of the *i*th mode (e.g., not at all, a little, somewhat, much, very much); *π_i_* represents the weight assigned to the *i*th mode (e.g., 1 = not at all, 2 = a little, 3 = somewhat, 4 = much, 5 = very much); *N* represents the total number of observations (i.e., the total number of respondents); *k* represents the number of points (in this case = 5) in the Likert scale. The AI value ranges between zero and one: the closer the value to one, the greater the annoyance.

As regards AI, since the normality test indicated that the distribution was not normal (*p* < 0.05), non-parametric statistical tests were used. In order to evaluate the correlation of each question with the AI, the Spearman test was performed. A descriptive analysis of the AI distribution was conducted both of the total number of respondents and the sample stratified by area, age group, and gender. The differences in the AI distribution between areas, age groups and gender were tested by the Kruskal-Wallis non-parametric test in order to evaluate which of these factors influenced the AI.

The median AI was also calculated (MAI) for each classroom. The MAI validation was obtained by calculating a similar index by the Structural Equation Model (SEM-AI) using the same set of variables [45]. SEM is one of the most used methods in the analysis of behavioral data in consideration of its robustness. Starting from a set of variables, SEM allows to identify one or more informative indicators. The goodness of fit of the SEM-AI was very good (standardized root mean squared residual, SRMSR < 0.10). The MAI distribution was very similar to SEM-AI distribution (correlation between MAI and SEM-AI was very high: ρ = 0.96), confirming the validity of the MAI. Finally, the simpler and more comparable MAI was used.

### 2.3. Measurements of Noise Data

The procedure for the acoustical evaluation of each classroom is presented in [43]. Measurement campaigns were carried out in the schools and a Global Noise Score (GNS) was assigned to each classroom, with a related score range. The GNS was obtained by summing the single scores assigned to a set of six parameters, defined in accordance with international standards:

The L_DAY_ (average hourly noise equivalent level during daytime according to Italian law, 6 a.m.–10 p.m.) to investigate the exposure to external sources, calculated by:

1. External noise monitoring (L_eq-ext_); In schools in Italy, limits depend on acoustic zoning, however a limit of 55 dB (A) is recommended, when 50 dB (A) is not possible [46].
2. Internal short-term measurements (L_eq-int_); As the internal noise level with open windows (L_DAY−int_) is not fixed by the regulation, this reference was set to 5 dB (A), which is less than the corresponding class for the external level (i.e., 50 dB (A) when the L_eq-ext_ is 55 dB (A)).

The following four parameters are used to investigate the acoustical characteristics of buildings:

3. Façade insulation: D_2m,nT,w_, calculated as the difference between internal and external noise measured at a distance of 2 m from the façade and corrected by reverberation time.
4. Wall insulation: R’_w_; A national specific decree establishes the limits for the façade isolation parameter D_2m,nT,w_ and for the wall isolation parameter R’_w_. For school buildings D_2m,nT,w_ should be more than 48 dB and R’_w_ more than 50 dB [47].
5. Reverberation time: RT; The scientific community has set the highest value of reverberation time at RT = 0.8 s (the value is intended for medium small classes, for a complete guidance as function of volume see [47]).
6. Speech intelligibility index: STI. No limits have been fixed for STI, but a scale of reasonable values according to the international standard is available [48] and can be used to evaluate the results.

The GNS ranges from 6, corresponding to the simultaneous minimum score for all the parameters—attributed on the basis of the deviation from the limits considered—to 30, when each parameter has the highest score. For further details, see the article by Chetoni et al. [43]. Thus, the higher the GNS, the better the acoustical environment of the classroom. In the present study, the range of parameters have been subdivided into equidistant intervals.

### 2.4. Measurement Protocol

No railways or airports are located in the proximity of the examined schools and the main noise source is always road traffic. In Naples noise is also due to the anthropic noise from markets and public spaces, whilst in Taranto, there are some industrial sources close to the schools.

The external noise level was acquired through a 7-day long measurement programme, with the microphone placed 1 m from the main outside façade of the school, at a height of 4 m [49]. All short internal measurements were performed in the afternoon, without the students. The internal noise level was obtained with short-term measurements of a duration of 30 min with open windows, taking the average values from two different positions: the center of the room, assumed to be the average position of a student, and 1 m from the windows, which is the worst listening position. All the noise measurements were carried out with class 1 spectrum analyzers. In the schools we monitored, windows are generally open during the lessons, due to the warm average climate and to general classroom overcrowding. Therefore, according to the guidelines of the Environmental Protection Agency-Tuscany Region [50], a correction factor was applied to the short-term noise level to evaluate the average daytime noise level inside the classroom (L_eq−Int_). The procedure assumes that the internal and external noise levels have the same time evolution. 30 min is not a sufficient amount of time for evaluating long-term internal level. Thus, the correction takes into account the daytime evolution and correct the afternoon level, providing a number representative of the whole daytime.

In addition, the daily outside noise level (L_eq−Ext_) was obtained for those classrooms that were not facing the main road and where the external measurement was performed. L_eq−Ext_ was calculated from the long-term external measurement on the main road and the internal long-term measurement calculated as explained above from measurements in each classroom. The attenuation term *A* from the outside measurement and the internal one of the classroom facing it was considered to be the same for all the outside walls. Thus, the external noise levels of the classrooms of back external façades were calculated by adding the term *A* to the corresponding internal noise levels. The correction can be applied only for noise with the same spectrum, which is almost what happens in all the surveyed locations due to the predominant road traffic.

**Figure 1:**
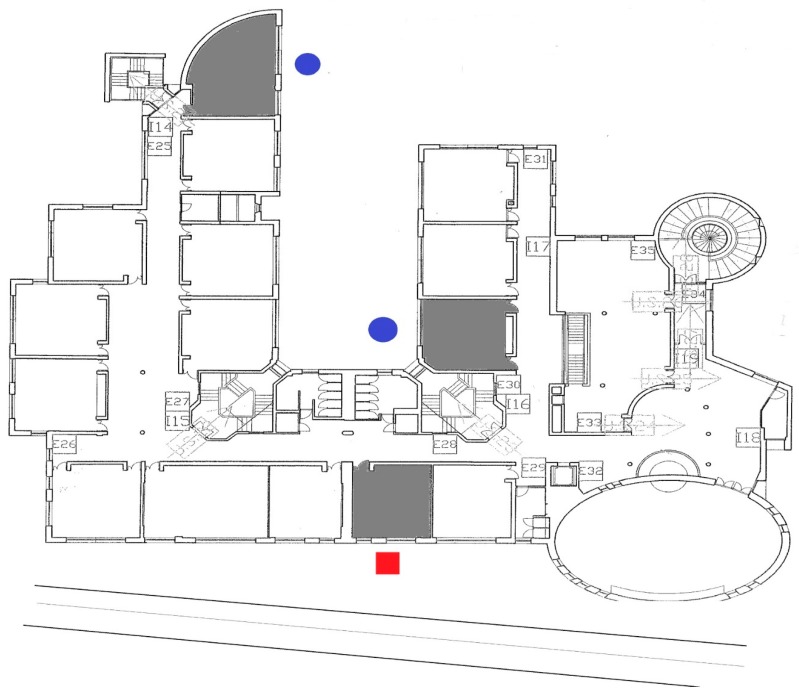
Example of school measurement positions. The measured classrooms are filled and the red square corresponds to external long-term measurement positions. As explained above, external levels are reported in blue circles.

Figure 1 shows a map of one of the surveyed schools. The measured classrooms are filled and the red square corresponds to the external long-term measurement position. As explained above, external levels are reported in blue circles.

After the characterization of environmental noise, the facade insulation had to be tested. The interior sound quality is due to the external sources and the building characteristics [51,52], therefore the standardized level difference D_2m,nT_ parameter was monitored using a wide-band pink noise generated by an emitter located outside the school in front of the measured room. The parameter represents the room’s insulation from the external noise with closed windows. The R parameter, calculated by means of similar measurements, represents the insulation between the adjacent rooms and the corridors through. Finally, the acoustic characteristics of the rooms were evaluated: reverberation time and STI index were measured using an MLS signal.

### 2.5. Correlation between MAI and GNS

The distributions of MAI and noise data by mean, standard deviation, 25th percentile, median, 75th percentile, 95th percentile, minimum and maximum were measured. A Shapiro-Wilk normality test was applied to verify that MAI and noise measurements were not normally distributed. The correlation between MAI and noise measured in each classroom was then performed by the non-parametric Spearman test [53]. In order to correlate perceived and measured noise, the correlation factor ρ and the *p* value were calculated between GNS and MAI, in addition to all correlations between the distribution of single annoyance variables and the distribution of measured noise parameters. The criteria assigned in order to determine the goodness of correlation were the following:

- ρ > |0.5| and *p* < 0.05, very good correlation;
- ρ < |0.5| and *p* < 0.05, good correlation;
- ρ < |0.5| and 0.05 < *p* < 0.1, borderline correlations;
- ρ < |0.5| and *p* ≥ 0.1, no statistically significant correlation.

Statistical significance was set at *p* < 0.05 (two-tailed). All the analyses were performed using STATA version 13.0 (StataCorp., College Station, TX, USA).

## 3. Results

Of the 521 students included in the study, a total 503 questionnaires were returned (297 males; age 14.1 ± 2.2 years [mean ± SD]). Overall, 67% of students aged 14+ years, compared to students aged <14 years, thought that their school was not/a little noisy (*p* < 0.001) with significant differences also found within gender (younger vs. older male group: 51.8% vs. 48.2% respectively, *p* < 0.01; younger vs. older female group: 31.9% vs. 68.1% respectively, *p* < 0.001).

As shown in Table 2, all responses of the students to the individual questions used to generate AI had a very good correlation with the index. Annoyance (question b—“How annoying is the noise you usually hear when you’re at school?”) was the highest contributor to annoyance, whereas a poor listening environment was the least (question c1—“I cannot hear when people are speaking in the room”) reported.

The mean AI was significantly (*p* < 0.01) higher in industrialized areas (Taranto and Naples), compared to the other two areas. Statically significant differences were found between age groups (*p* < 0.001), with noise most perceived in their own school by younger students. No significant differences of AI by gender were recorded (Table 3).

Descriptive statistics of AI are reported in Table 4. In the overall sample, individual AI scores ranged from 0.20 to 0.84 with a mean of 0.46. A similar variability was reported considering each variable (range 0.20–0.84).

Classroom MAI scores ranged between 0.32 and 0.64. The percentage of students who on average reported that they could not hear when people were speaking in the classroom was only 15%, although the mean percentage of students who could not concentrate due to noise increased to 47%. Responses of students to questions a, b, and d had a score approximately at the middle point of the Likert scale. Annoyance varied between “a little” and “a lot”, showing a high variability in the sample (Table 4).

The scores of the GNS indicator ranged between 7.00 and 21.00, with a mean of 12.07. The worst contribution to GNS was noise from external sources, facade insulation, and reverberation time. For only 25% of the classrooms, the external measured noise had values less than 54 dB (A), which was slightly below the national regulation limit. The maximum value reached by façade insulation was 43 dB (A), with a mean of 27.3 dB (A) that is much lower the national limit of 48 dB (A), i.e., rooms are very poorly isolated from noise coming from outside. Reverberation time is the time required for the sound to “fade away” in a closed area, in particular it measures the time needed to the sound to decrease its energy of 60 dB (A). All classrooms had a reverberation time that was above the guide limit of 0.8 s. Reverberation had in turn a negative impact on STI scores which ranged between 0.4 and 0.68 in the classrooms, thus the best measured value is far from the lowest limit of class international standard (0.75–1.00) (Table 5).

**Figure 2:**
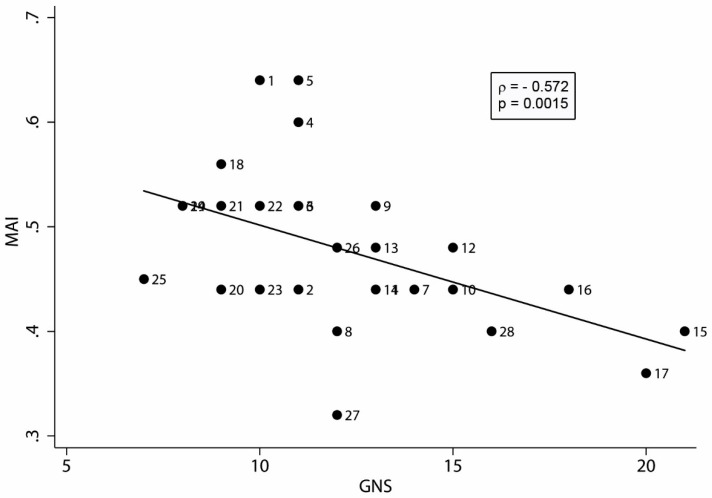
Relationship between GNS and MAI for all classrooms (*n* = 28). The fitted regression line is shown. Each classroom was identified by a number. GNS: Global Noise Score; MAI: classroom Median Annoyance Index.

The graph in Figure 2 shows the negative correlation between MAI and GNS considering data collected in each classroom. As specified in [43], noise measurements were performed in three classrooms for each school. Thus, as the measures were conducted on 24 classes out of the total 28, for the four missing measures the experts of the GIOCONDA working group attributed the noise data of classrooms of the same school that had similar structural characteristics and position.

According to the criterion described in Section 2.5, MAI and GNS showed a very good negative correlation (ρ = −0.572 with 0.0015 statistical significance). The decrease in MAI with the increase in the acoustic quality of the classroom, means that for higher GNS (i.e., lower background noise and lower reverberation time) the noise and annoyance perceived were lower.

Thus, where there are better acoustical conditions, the reported annoyance decreases. The Spearman Rank-order coefficient is considered appropriate to test the correlation between MAI and GNS because the smoothing analyses generally show monotonic relationships (Supplementary File). In the few cases where loss of monotonicity was observed at the extremes of the distribution, this was based on 2–3 points for each correlation analysis (equal to about 7–11% of the sample), nonetheless do not affect the results. In fact, excluding these points the results obtained were similar or more significant s (i.e., ρ between MAI and GNS = −0.6089, *p* = 0.0012).

In addition, performing a sensitivity analysis considering only classrooms in which noise was measured (*n* = 24), the correlation between MAI and GNS was similar to our results (ρ = −0.5821; *p* = 0.0045).

MAI showed a very good correlation with L_eq_ext_, L_eq_int_ and RT, and a borderline correlation with STI (Table 6). Conversely, annoyance was not influenced by façade insulation as well as wall insulation.

With regard to the response to question a (“Do you think your school is noisy?”), a good correlation was found with the global index, the noise measured outside the school, and RT and a very good correlation with noise measured inside the classroom.

Annoyance (response to questions b “How annoying is the noise you usually hear when you’re at school?”) showed a very good correlation with L_eq_ext_ and a good correlation with GNS, L_eq_int_, and RT.

Response to question c1 (“I cannot hear when people are speaking in the room”) correlates (without statistical significance) only with noise levels L_eq_int_ and L_eq_ext_, whereas response to question d (“How often do you notice noise?”) was related to all noise parameters, except the two concerning the structural characteristics of the classroom. Question c2 (“The noises distract me”) showed no correlation with any noise parameter.

## 4. Discussion

The aim of this study was to investigate the degree of annoyance of students aged between 11 and 18 years in their schools and how it is correlated with actual noise measurements. Studies on risk perception, including children’s own perception of noise, are essential background information for risk communication strategies [9]. Questionnaires are the preferred instrument to determine noise perception, annoyance, disturbance, difficulty in listening and speech comprehension of both students and teachers [29,54,55]. Previous research performed via questionnaires has revealed that children are sensitive judges of their noise environments, discriminating the amount and types of noise sources to which they are exposed [29,54].

The Annoyance Index used and validated in this paper summarizes part of the information collected through a questionnaire. Specifically, AI is the result of weighted average scores of answers to questionnaire items, estimating both the individual perceived noise exposure and annoyance.

Overall, the mean of AI (0.46) suggests that annoyance of students is modest in comparison to the measured noise. This finding is consistent with those reported in two qualitative studies in which the perceived risk of noise pollution as a hazard was minimal in children aged 10–13 [56], and the judgment of environmental noise was ranked as a medium-level risk factor in students aged 15–17 [38].

The subsequent step was to assess how AI was associated with other variables, such as geographical area, age and gender. The variables potentially influencing the AI were study area and age. An individual’s ability to perceive environmental risks is proportional to their own sensitivity to environmental problems [38]. In Naples and Taranto, the most industrialized and polluted cities of the four areas examined, the students’ judgment of noise was higher, possibly because environmental issues are a hot topic in their city. Compared to these areas, the other areas have smaller populations, less industry, less environmental noise and consequently fewer adverse effects.

Significantly higher levels of annoyance were found among younger children (*p* < 0.001). These findings corroborate results described in other investigations [26,27,28]. In our study, no difference in judgment of noise was reported by gender. In contrast, among the >14 year-old students, males showed a higher annoyance in all the areas, as reported in a Turkish study [38], though another investigation carried out on students from the University of British Columbia highlighted that women showed slightly higher scores [57].

The classroom MAI showed a very good reverse correlation with GNS, the global score obtained by summing up the six parameters characterizing the acoustical environment of classrooms. In particular, perceived noise correlated highly with external sources, considering that L_eq_ext_ is probably associated with noise at the entrance and on leaving the school, and transportation represents the greatest source of noise, as previously reported [29,54,55]. L_eq_int_ can be largely influenced by the insulation characteristics of classrooms. Acoustical quality in classrooms is further dependent on voice reverberation, thus shorter reverberation times contribute to an environment in which listening and, as a consequence, learning is facilitated, and speech intelligibility is maximized [55,58,59,60]. The good maintenance of windows and doors could improve the insulation [61], which was in fact lacking in almost all the measured rooms. Furthermore, it has been demonstrated that false ceilings improve RT values, which appears to be the most serious problem related to the learning process [27].

The design and quality of school buildings differ not only among schools but also within the same school and these variations influence the types and intensity of noise that students are exposed to [39]. In our study, the GNS indicator had a mean value of 12.07, highlighting the unsatisfactory acoustical conditions of the classrooms examined. In fact, according to its definition, it would have been sufficient with values over 16. External noise sources, façade insulation, and reverberation time provided the most negative contribution to GNS. A recent study considering more than one hundred façades of Italian schools of all levels (from nursery to upper secondary school) and built in different time periods and building technologies, revealed that almost all the schools needed sound insulation refurbishment and acoustic treatment of classrooms [62]. Thus, the schools selected within the project across the country can be considered as representative of the general conditions in Italian schools.

Due to the good correlations of some parameters (L_eq_ext_, internal noise, and reverberation time) GNS as a whole has been found to be associated with annoyance. External L (Amax) available from L_eq_ext_ levels have been shown to be a significant factor in the reported annoyance by both 6–11 year-olds [54] and secondary school students [55].

It is worth noting that the parameters D_2m,nT,w_ and R’_w_ were not correlated with annoyance evaluated by MAI. This is probably due to the fact that they are all below the law requirements and with almost the same values. MAI is influenced by several other parameters and presents a larger spread within classes. Thus, it is easier for students to associate perceived sound levels with external sources than to the poor insulation of the school building. STI was not highly correlated with annoyance, suggesting that in actual classroom situations, in addition to the physical acoustical characteristics of the classroom, a number of factors play an important role in students’ subjective judgment of the environmental listening quality (e.g., age, gender, moderate to severe hearing impairment), as already described by Kennedy et al. [57].

As the principal aim of this study was to investigate the relationship of MAI with noise measurements in classrooms, the questionnaire concerned the general noise judgment students had of their schools without considering the specific sources of noise. A previous work developed a “perceived listening ease” (PLE) score that was a measure of perceived classroom listening quality during typical classroom use, and was calculated by summing the scores of questionnaire responses standardized in a five-point scale [57]. In agreement with [57], we found that a simple additive approach to obtain AI gave similar results to weighted average scores. As regards individual AI, since the normality test indicated that the distribution was not normal (*p* < 0.05), MAI was estimated by non-parametric regression, in contrast to class-average PLE scores.

Despite being aware of the limitation represented by the small sample size, we believe that the judgment profiles, which were different according to age and geographical areas, represent a useful starting point. The reliability of the AI can be further improved by extending the research to a larger sample of schools (in differently characterized areas), and by evaluating the role of other variables that could influence the noise-related risk perception, such as socio-economic and ecological determinants. In addition, it should also be highlighted that noise-related judgment is part of a wider assessment of young people’s judgment of risk associated with environmental pollution. A further limitation is that we did not consider the awareness of the same environmental stressor at home.

Finally, the efficiency of global indicators formulated for noise mitigation purposes has been reported in several papers [63,64]. Thus, easily usable indicators can facilitate the communication of measurement outcomes to stakeholders (teachers, families and policy makers) and thus lead to the actions needed. GNS was developed in order to find an easy indicator related to annoyance and quality, without claiming it as an absolute reference which would obviously depend on national requirements. Besides its definition, a correlation between noise parameters and MAI was found. This will thus ensure that by improving noise parameters, judgment will also improve, driving policy actions to better noise quality in schools.

## 5. Conclusions

Previous studies have shown that a poor acoustical environment in classrooms, which influences children’s speech listening and comprehension, potentially interferes with their educational performance, since effective listening is vital to school learning. Our results confirm that annoyance represents the most widespread subjective response to noise, and noise judgment and the consequent disturbance are highly correlated with acoustical classroom conditions.

Despite the recent interest in achieving improved acoustical quality in school environments, many classrooms still do not satisfy international and national standards concerning ambient noise levels and reverberation. The findings reported in this paper corroborate that noise in schools is a serious issue, which should not be neglected by local administrations. In fact, all the classrooms exceed the limits established by the Italian law in terms of external noise and building noise. As we showed, the eight schools examined in this study, located in different areas of the country, can represents the average Italian scenario.

The responses to the questionnaires (investigating not only the awareness about noise here presented, but also air pollution, risks to health of children and the community, and behaviors related to environmental protection) were discussed with students together with the data of environmental monitoring and the results of a collection of information about local issues in the environment and health domain. This process of learning, discussion and elaboration at school produced a “challenge booklet” [65] including all the results, further elaborated in “recommendations to local decision-makers” [66], composed by each school and presented during a public “engagement event” [66]. All the eight documents started saying: “Dear decision makers, we recommend …”, and the request related to noise, summarized from the documents (available in Italian at the GIOCONDA project website) were related to three main topics. First school improvement: protection of walls in the classroom (that was realized in two schools using insulating panels) restoration and replacement of windows. Second transport policies: to improve public transport; to reduce car traffic; to promote electric cars; to block streets close to schools during entrance and exit time; to avoid motorcycle circulation in school surroundings. Third, more general, related to the participation of students: in decisions, in monitoring actions, in evaluation results through the establishment of a “Young Citizen’s Municipal Council”. As first application of GIOCONDA methodology the results of the public discussions based on the measured data has been included in the training plans of the Centers of Education for Sustainability in the cities of Ravenna, Ferrara and some municipalities of the Lower Valdarno. The recommendations produced by the classes of Ravenna were included in the master plan for the sustainable mobility of the city in 2016.

The GIOCONDA project carried out a pilot study in four Italian areas, and implemented a web platform to be used by other schools and local authorities to highlight problems, discuss ideas and find solutions regarding environmental problems. The characterization of annoyance is an important step in increasing the social awareness regarding the reduction of environmental risks. Children spend long periods of time at school, and are therefore the most vulnerable subjects to environmental pressures. Their judgment of environmental stressors is an important indicator to improve their own environment and a means to understanding the judgments, attitudes, concerns, and wishes of the entire community. The results provided by this study and the tools for sharing knowledge and data could contribute to raising awareness of environmental health issues and improving health prevention and protection. GNS was developed in order to find an easy indicator related to noise judgment and quality, without claiming it as an absolute reference, which would obviously depend on national requirements. Besides its definition, a correlation between noise parameters and MAI was found. This will thus ensure that by improving noise parameters, judgment will also improve, driving policy actions to better noise quality in schools. On the other hand, MAI can be a first filter to select schools to be monitored and to address actions, being that MAI is related to real noisiness of schools.

## Figures and Tables

**Table 1:** Description of noise related-questions.

Question ID	Noise-Related-Questions	Variables Scale	Scale Labels
a	Do you think your school is noisy?	Likert-scale	1 = not at all 2 = a little 3 = somewhat 4 = a lot 5 = very much
b	How annoying is the noise you usually hear when you’re at school?	Likert-scale (1–5)	1 = not at all 2 = a little 3 = somewhat 4 = a lot 5 = very much
c1	Is annoying noise in the area around your school causing you any problems? I cannot hear when people are speaking in the room.	Dichotomous (0–1)	1 = yes 0 = no
c2	Is annoying noise in the area around your school causing you any problems? The noise distracts me.	Dichotomous (0–1)	1 = yes 0 = no
d	How often do you notice that there is noise?	Lykert-scale (1–5)	1 = never 2 = seldom 3 = sometimes 4 = often 5 = always

**Table 2:** Correlations between individual AI and questionnaire items.

Index	Correlation Coefficient	a	b	c1	c2	d
AI	ρ	0.5739	0.7726	0.4405	0.5562	0.5996
	*p*	<0.001	<0.001	<0.001	<0.001	<0.001

Correlations were performed by non-parametric Spearman test. ρ: correlation coefficient; *p*: observed probability; a, b, c1, c2, d: noise-related questions, AI: Annoyance Index.

**Table 3:** Mean (±standard deviation) and percentile values of annoyance index in the overall sample and by area, age group, and gender.

Factor	N	Mean ± SD	25th *p*	50th *p*	75th *p*	95th *p*	min	max	*p*
Naples	128	0.49 ± 0.12	0.40	0.48	0.58	0.68	0.24	0.76	0.0027
Ravenna	153	0.45 ± 0.10	0.36	0.44	0.52	0.64	0.24	0.76	
Taranto	102	0.48 ± 0.11	0.40	0.48	0.56	0.64	0.24	0.84	
Lower Valdarno Valley	120	0.44 ± 0.14	0.34	0.44	0.52	0.70	0.20	0.84	
<14 years	287	0.49 ± 0.12	0.40	0.48	0.60	0.68	0.24	0.84	0.0001
14+ years	216	0.42 ± 0.11	0.36	0.42	0.52	0.60	0.20	0.84	
Female	206	0.47 ± 0.11	0.40	0.45	0.56	0.68	0.24	0.76	0.1158
Male	297	0.46 ± 0.12	0.36	0.44	0.56	0.68	0.20	0.84	
Total	503	0.46 ± 0.12	0.36	0.44	0.46	0,68	0.20	0.84	

SD: standard deviation; 25th *p*, 50th *p*, 75th *p*, 95th *p* represent the first quartile, median, third quartile, and 95th percentile, respectively; *p*: observed probability to evaluate the null hypothesis (no differences by area, age, and gender).

**Table 4:** Annoyance index—Descriptive of classroom-median AI (MAI) and questionnaire items distribution by classroom (*n* = 28).

Index Response	Mean ± SD	25th *p*	50th *p*	75th *p*	95th *p*	min	max
MAI (range 0–1)	0.48 ± 0.08	0.44	0.47	0.52	0.60	0.32	0.64
a (range 1–5)	2.88 ± 0.45	2.50	2.88	3.18	3.45	1.94	3.92
b (range 1–5)	2.70 ± 0.57	2.51	2.64	2.99	3.82	1.48	4.00
d (range 1–5)	3.13 ± 0.43	2.90	3.07	3.37	3.91	2.32	4.14
c1 (%)	15 ± 14	5	10	26	43	0	45
c2 (%)	47 ± 16	3.4	46	63	69	16	70

SD: standard deviation; 25th *p*, 50th *p*, 75th *p*, 95th *p* represent the first quartile, the median, the third quartile, 95th percentile, respectively; a, b, c1, c2, d: noise related questions; MAI: classroom median Annoyance Index.

**Table 5:** Noise measured index—GNS and single noise parameter distribution by classroom (*n* = 28).

Index	Mean ± SD	25th *p*	50th *p*	75th *p*	95th *p*	min	max
GNS (range 6–30)	12.07 ± 3.38	10.00	11.00	13.00	20.00	7.00	21.00
L_eq_ext_ (dB)	60.26 ± 8.27	54.00	61.40	64.40	71.00	37.20	73.50
L_eq_int_ (dB)	47.39 ± 10.62	37.90	48.45	56.70	60.10	23.10	62.60
D_2m,nT,w_ (dB)	27.33 ± 5.65	24.00	28.00	31.00	35.00	15.00	43.00
R’_w_ (dB)	38.95 ± 8.66	31.00	42.00	44.00	49.00	21.00	49.00
RT (s)	1.91 ± 0.50	1.58	1.92	2.36	2.84	0.88	2.88
STI (s)	0.51 ± 0.06	0.48	0.49	0.53	0.66	0.40	0.68

SD: standard deviation; 25th *p*, 50th *p*, 75th *p*, 95th *p* represent the first quartile, the median, the third quartile, 95th percentile, respectively; GNS: Global Noise Score; L_eq_ext_: external noise monitoring L_eq_int_: internal short-term measurements; D_2m,nT,w_: façade insulation; R’_w_: wall insulation; RT: reverberation time; STI: speech intelligibility index.

**Table 6:** Correlations between perceived noise and measured noise.

Index	MAI (Range 0–1)	a—“Do You Think Your School Is Noisy?” (Range 1–5)	b—“How Annoying Is the Noise You Usually Hear When You’re at School?” (Range 1–5)	c1—“I Cannot Hear When People Are Speaking in the Room.” (%)	c2—“The Noise Distracts Me.” (%)	d—“How Often Do You Notice That There Is Noise?” (Range 1–5)
GNS (range 6–30)	**−0.572** **0.0015**	−0.425 0.0241	−0.4045 0.0327	−0.292 0.1311	−0.2098 0.2838	−0.4276 0.0232
L_eq_ext_ (dB)	0.6113 0.0005	0.4507 0.0161	0.6051 0.0006	0.3357 0.0807	0.1446 0.4628	0.5883 0.001
L_eq_int_ (dB)	0.6051 0.0006	0.5298 0.0037	0.437 0.02	0.3723 0.0511	0.0657 0.7399	0.3731 0.0505
D_2m,nT,w_ (dB)	0.0912 0.6443	0.0432 0.8274	0.2555 0.1895	0.0276 0.8891	0.0554 0.7796	0.1491 0.4488
R’_w_ (dB)	0.1715 0.3829	0.2542 0.1918	0.1513 0.4422	−0.0338 0.8644	−0.1674 0.3945	0.1108 0.5745
RT (s)	0.5122 0.0053	0.4031 0.0334	0.4201 0.026	0.3018 0.1186	0.0568 0.7741	0.465 0.0127
STI (s)	−0.3303 0.086	−0.1465 0.4568	−0.228 0.2423	−0.2759 0.1553	−0.0882 0.6552	−0.3651 0.0561

Each cell contains the correlation factor (ρ) and the *p* value (observed probability). Very good and good correlations are highlighted in dark grey and grey, respectively; borderline correlations are highlighted in light grey. GNS: global noise score; MAI: classroom-median AI; L_eq_ext_: external noise monitoring; L_eq_int_: internal short-term measurements; D_2m,nT,w_: façade insulation; R’_w_: wall insulation; RT: reverberation time; STI: speech intelligibility index; a, b, c1, c2, d: noise-related questions.

## Supplementary Materials

The following are available online at http://www.mdpi.com/1660-4601/15/2/208/s1, Supplementary File: Smoothing correlations by LOWESS (locally weighted scatterplot smoothing) and LPOLY (Kernel-weighted local polynomial smoothing).
