# Supplementary material for: Annoyance Judgment and Measurements of Environmental Noise: A Focus on Italian Secondary Schools
Source: Int J Environ Res Public Health. 2018 Jan 26;15(2):208. doi: 10.3390/ijerph15020208 (PMC5858277; doi:10.3390/ijerph15020208)
Supplement: Supplementary file 1 [file ijerph-15-00208-s001.zip › ijerph-254179-Supplementary file.docx]

**Supplementary Materials: Annoyance Judgment and Measurements of Environmental Noise: A Focus on Italian Secondary Schools**

**Fabrizio Minichilli, Francesca Gorini, Elena Ascari, Fabrizio Bianchi, Alessio Coi, Luca Fredianelli, Gaetano Licitra, Federica Manzoli, Lorena Mezzasalma and Liliana Cori**

**Supplementary File.** Smoothing correlations by LOWESS (locally weighted scatterplot smoothing) and LPOLY (Kernel-weighted local polynomial smoothing) referring to Table 6.

|  | **GNS** | **Leq_ext (dB)** | **Leq_int (dB)** | **RT (s)** | **STI (s)** |
| --- | --- | --- | --- | --- | --- |
| **MAI *Range (0–1)*** |  |  |  |  |  |
| **A—“Do you think your school is noisy”? *Range (1–5)*** |  |  |  |  |  |
| **b—“How annoying is the noise you usually hear when you’re at school?” *Range (1–5)*** |  |  |  |  |  |
| **d—“How often do you notice that there is noise?”*Range (1–5)*** |  |  |  |  |  |

Note. Only significant correlations are shown.

**Supplementary File.** Smoothing correlations by LOWESS (locally weighted scatterplot smoothing) and LPOLY (Kernel-weighted local polynomial smoothing) referring to Table 2.

|  | **a—“Do you think your school is noisy?” *Range (1–5)*** | **b—“How annoying is the noise you usually hear when you're at school?” *Range (1–5)*** | **c1—“I cannot hear when people are speaking in the room.” *%*** | **c2—The noise distracts me. *%*** | **d—“How often do you notice that there is noise?” *Range (1–5)*** |
| --- | --- | --- | --- | --- | --- |
| **MAI *Rang***  ***(0–1)*** |  |  |  |  |  |
