# Supplementary material for: Annoyance Judgment and Measurements of Environmental Noise: A Focus on Italian Secondary Schools
Source: Int J Environ Res Public Health. 2018 Jan 26;15(2):208. doi: 10.3390/ijerph15020208 (PMC5858277; doi:10.3390/ijerph15020208)
Supplement: Supplementary file 1 [file ijerph-15-00208-s001.zip › ijerph-254179-Supplementary file.pdf]

Supplementary Materials: Annoyance Judgment and Measurements of Environmental Noise: A Focus on Italian Secondary Schools

Fabrizio Minichilli, Francesca Gorini, Elena Ascari, Fabrizio Bianchi, Alessio Coi, Luca Fredianelli, Gaetano Licitra, Federica Manzoli, Lorena Mezzasalma and Liliana Cori

Supplementary File. Smoothing correlations by LOWESS (locally weighted scatterplot smoothing) and LPOLY (Kernel-weighted local polynomial smoothing) referring to Table 6.

	GNS	Leq_ext (dB)	Leq_int (dB)	RT (s)	STI (s)
MAI *Range (0–1)*	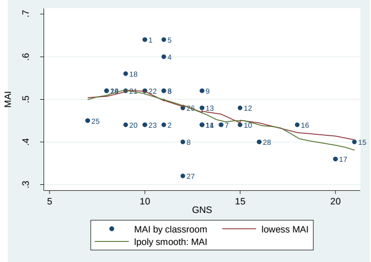	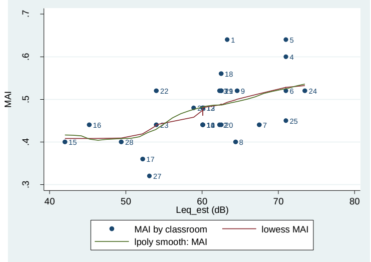	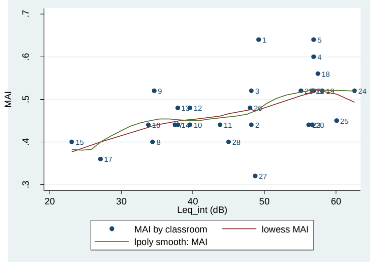	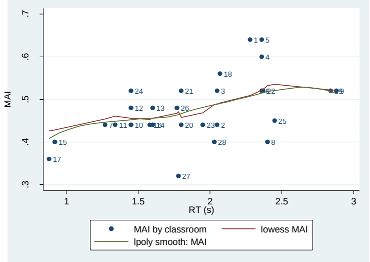	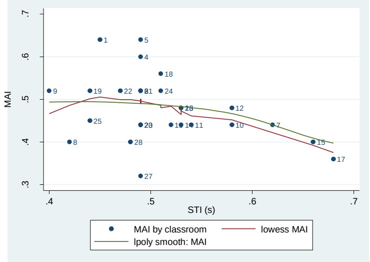
A—“Do you think your school is noisy”? *Range (1–5)*	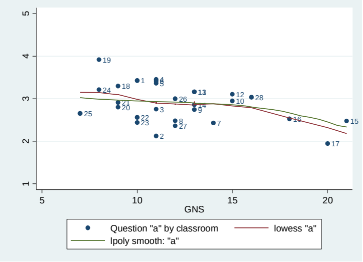	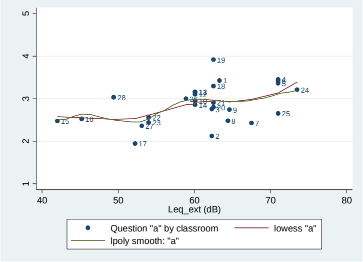	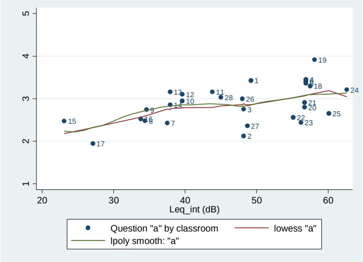	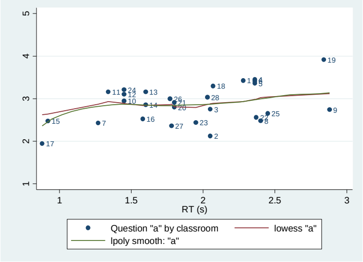	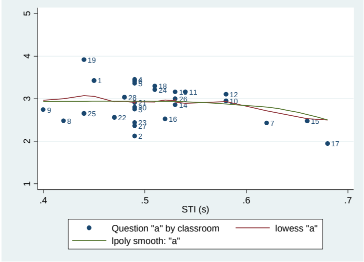
b—“How annoying is the noise you usually hear when you’re at school?” *Range (1–5)*	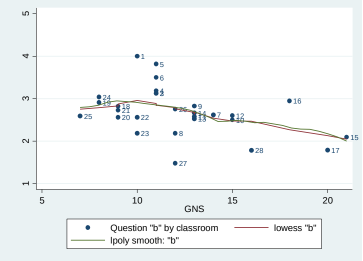	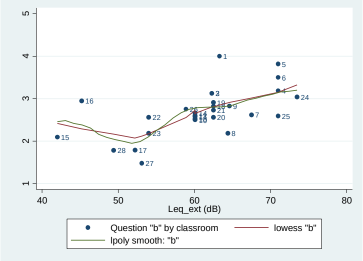	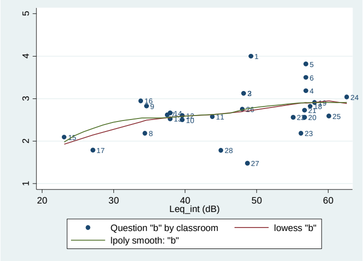	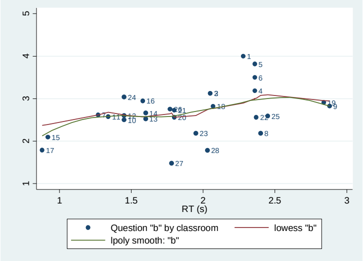	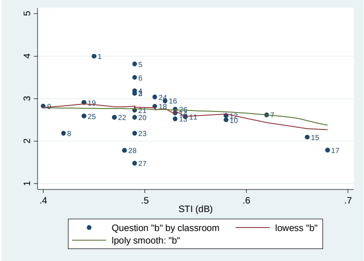

**d—“How often do you notice that there is noise?”*Range (1–5)***	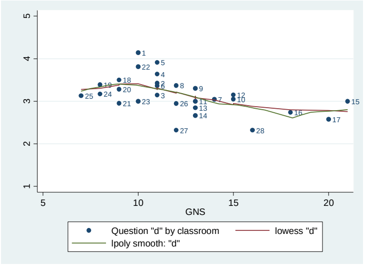	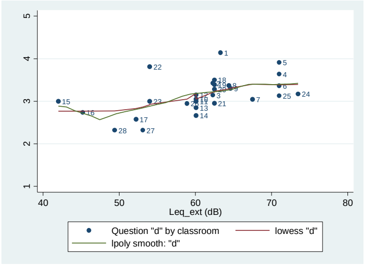	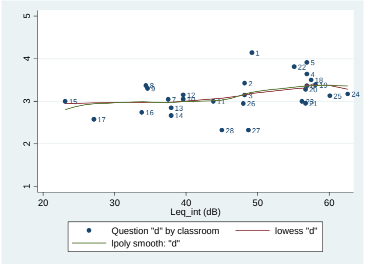	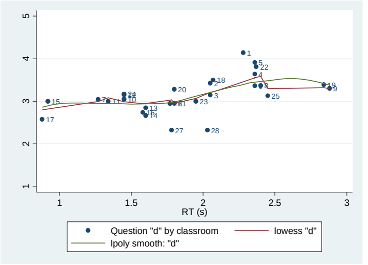	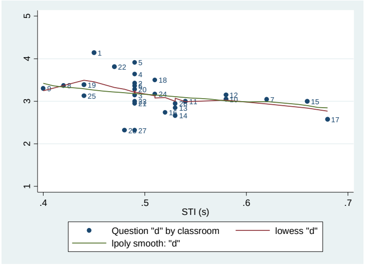

Note. Only significant correlations are shown.

Supplementary File. Smoothing correlations by LOWESS (locally weighted scatterplot smoothing) and LPOLY (Kernel-weighted local polynomial smoothing) referring to Table 2.

	**a—“Do you think your school is noisy?” *Range (1–5)***	**b—“How annoying is the noise you usually hear when you're at school?” *Range (1–5)***	**c1—“I cannot hear when people are speaking in the room.” %**	**c2—The noise distracts me. %**	**d—“How often do you notice that there is noise?” *Range (1–5)***
**MAI** *Rang (0–1)*	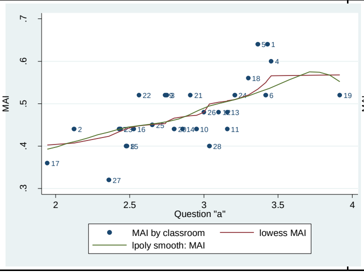	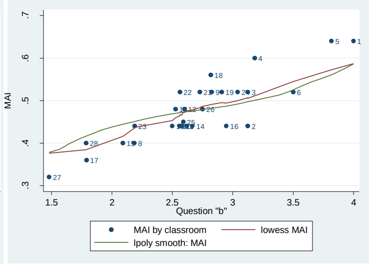	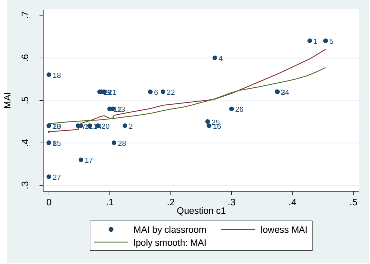	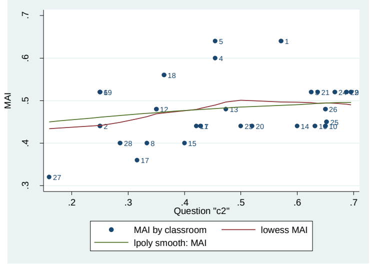	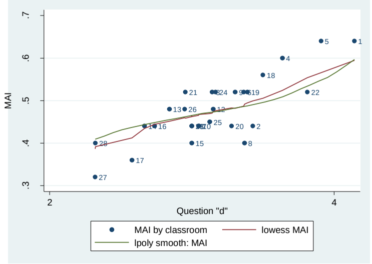
